# Symptomatic bone marrow metastases in breast cancer: A retrospective cohort study

**DOI:** 10.3389/fonc.2022.1042773

**Published:** 2022-12-19

**Authors:** Ruohan Yang, Lin Jia, Guanyu Lu, Zheng Lv, Jiuwei Cui

**Affiliations:** Cancer Center, The First Hospital of Jilin University, Changchun, China

**Keywords:** breast cancer, bone marrow metastasis, prognosis, retrospective, cohort study

## Abstract

**Objective:**

Breast cancer symptomatic bone marrow metastasis (BMM) is rare and has a poor prognosis. Chemotherapy is usually the primary treatment, but it has limited efficacy, resulting in dose reduction and a decrease in quality of life due to the adverse effects of the agent. Other than chemotherapy, there are no other treatment studies for BMM. This study aimed to explore the clinicopathological characteristics of BMM patients with breast cancer, the prognosis using different treatment modalities, and the risk factors that affect the prognosis.

**Methods:**

This retrospective study included patients diagnosed with breast cancer BMM from January 2018 to January 2022 in the Cancer Center of the First Hospital of Jilin University. The analysis focused on the characteristics of the patients, the treatment regimen, and the prognosis.

**Results:**

Of 733 patients with advanced breast cancer, 33 patients were identified with BMM. All patients showed a hemoglobin decrease, and 25 (75.75%) presented with a fever of unknown origin. As for the metastasis breast cancer subtype, 25 (75.75%) were hormone receptor (HR) positive/human epidermal growth factor receptor 2 (HER2) negative, three (9.09%) had HER2 overexpression, and five (15.15%) were triple negative. The BMM patients had a median progression-free survival (PFS) of 7 months (1–21 months) and a median overall survival (OS) of 18 months (2–108 months). Among 25 HR^+^/HER2^−^ BMM patients treated with different modalities, the median OS of the endocrine therapy (ET) group was 23 months, compared with 5 months in the chemotherapy group. Cox proportional hazards models suggested that higher Eastern Cooperative Oncology Group (ECOG) scores and old age were associated with shorter survival.

**Conclusion:**

When breast cancer patients present with anemia and fever of unknown origin, BMM should be considered. For HR^+^/HER2^−^ patients with good physical status and can receive active treatment, CDK4/6 inhibitors combined with ET can be used to control disease progression, improve quality of life, and prolong survival.

## Introduction

Symptomatic bone marrow metastases (BMM) are the hematogenous spread of circulating tumor cells and the invasion of highly vascularized bone marrow. They manifested as hematopoietic function suppression, such as anemia, thrombocytopenia, and abnormal coagulation (1). Diffuse bone marrow involvement leading to profound cytopenias is rare in solid malignant tumors of nonhematologic diseases (2). Xiao et al. retrospectively analyzed 10,122 solid tumor bone marrow biopsy samples and found that lung, gastric, and breast cancer patients were prone to bone marrow infiltration (3). Although the specific mechanism of breast cancer BMM is not fully understood. It has been confirmed that bone marrow adipocytes (BMAs) and adipokines secreted by breast cancer cells are essential mediators in promoting breast cancer metastasis (4). BMAs secrete cytokines such as leptin, adiponectin, IL-1β, IL-6, TNF-α, and VEGF to promote breast cancer cell metastasis (5). BMAs can also release cytokines to activate dormant mesenchymal stem cells (MSCs) and cancer stem cells (CSCs) to increase their proliferation and promote breast cancer BMM (6).

Current literature suggests that chemotherapy prolongs survival in breast cancer patients with BMM (7, 8). Chemotherapy, however, can promote the growth of BMAs in the bone marrow, especially in the sacrum (9). Increased BMAs will further promote tumor cell escape and bone destruction around the tumor to promote tumor progression. At the same time, the adverse reactions of chemotherapy agents affected the patient’s quality of life (4). Kopp et al. found that the initial chemotherapy of BMM alleviated the patient’s cytopenias but did not significantly improve the patient’s prognosis (2). Only one case report highlights the positive role of anti-HER2 therapy in breast cancer BMM (10). Furthermore, there is a lack of large-scale studies on the prognosis of treatment regimens other than chemotherapy.

Therefore, we conducted a retrospective analysis based on our center’s cases to investigate the clinical characteristics of breast cancer BMM, the prognosis of patients with different treatment methods, and the risk factors affecting the prognosis.

## Methods

### Study population

Through the medical record system of our hospital, from January 2018 to January 2022, breast cancer BMM patients were retrospectively analyzed. The inclusion criteria were as follows:

1. Clinical manifestations are hypocytosis with or without fever of unknown origin; bone marrow aspiration biopsy confirmed cancer infiltration.
2. Patients who had complete medical records

The exclusion criteria included patients with primary tumors concurrently at other sites and those with metastases from which the tumor origin could not be determined.

### Data collection

Clinicopathological information was systematically extracted by reviewing medical records and included the following variables: hormone receptor (estrogen and progesterone) status, HER2 status, age at diagnosis of bone marrow metastases, disease stage at initial diagnosis, number of previous lines of treatment received, ECOG scores at diagnosis of BMM, regimens received, adverse events, time to disease progression, and time to death.

Endocrine therapy (ET) following the diagnosis of BMM includes aromatase inhibitors (AI) and cyclin-dependent kinase (CDK) 4/6 inhibitors.

### Statistical analysis

Survival was assessed using Kaplan–Meier analysis, and the log-rank test was used to determine overall survival (OS) rates between groups treated with different regimens. The Cox proportional hazards model was used to search for risk factors that affect OS in patients with bone marrow metastases. Progression-free survival (PFS) is defined as the time from the occurrence of BMM on any regimen to disease progression or death from any cause. OS was defined as the time from the occurrence of BMM receiving any treatment regimen to death.

Statistical significance was defined as a two-sided *p*-value of < 0.05. All statistical tests were performed using SPSS 23.0 (IBM Corporation Released 2013. IBM SPSS Statistics for Windows, Version 23.0, Armonk, NY, USA).

## Result

### Clinical features of patients with bone marrow metastases

**Figure 1:**
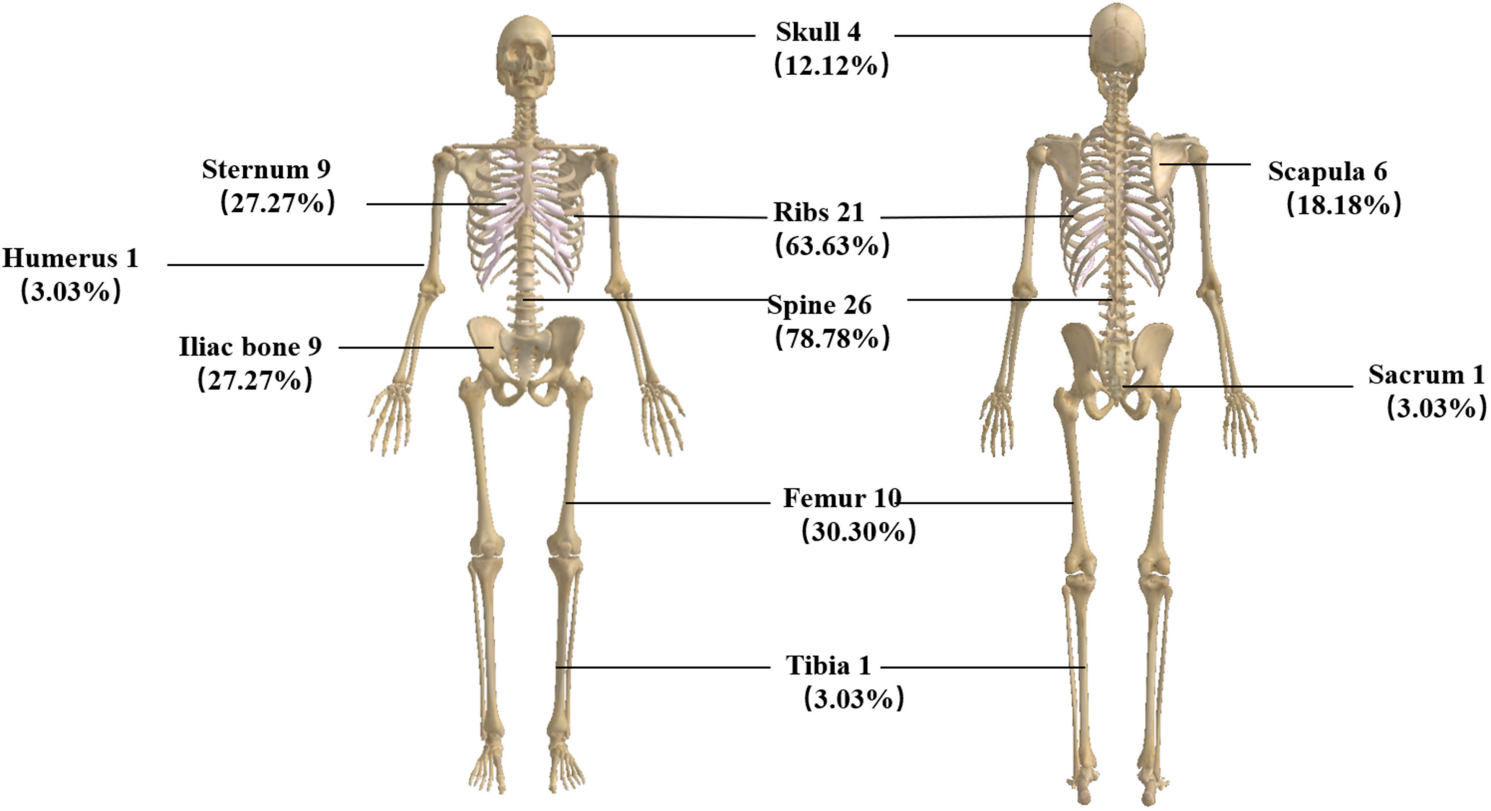
Pattern of bone metastases in patients with breast cancer BMM.

A total of 33 patients were included in this study. All denied a family history of breast cancer. The median age was 49.5 years (29–68 years); 25 (75.75%) were HR positive/HER2 negative, three (9.09%) had HER2 overexpression, and five (15.15%) were triple negative. As for metastasis breast cancer pathological type, 28 (84.85%) were invasive ductal carcinoma. In total, 28 patients (84.85%) had a high Ki-67 index expression, 14 (42.42%) were primarily diagnosed as *de novo*, and 15 (45.46%) had an ECOG score of 3. Clinically, all patients complained of fatigue, and 25 (75.75%) had fever with a negative etiological test. Blood routine revealed that all patients had a decrease in hemoglobin. Three of the cases (9.09%) showed thrombocytopenia, and two (6.25%) showed pancytopenia with no apparent cause. We also analyzed factors such as menstrual status, histological grade, and the number of previous lines of therapy (**Table 1**). All of the patients had bone metastases when they developed BMM. We analyzed the specific sites of bone metastases and found that the spine was the most common (78.78%), followed by the ribs (63.63%) and the femur (30.20%) (**Figure 1**).

**Table 1:** Clinical characteristics of patients with BMM.

Variable	*N* (%)
Age (years)	
Median age	49.5 (29–68)
≤60	25 (75.75%)
>60	7 (24.25%)
Menstrual status	
Postmenopause	11 (33.33%)
Premenopausal	22 (66.67%)
**Molecular typing**	
HR^+^/HER2^−^	25 (75.75%)
HER2 overexpression	3 (9.09%)
Triple negative	5 (15.15%)
Pathological type	
Invasive ductal carcinoma	28 (84.85%)
Invasive lobular carcinoma	2 (6.06%)
Others	3 (9.09%)
Ki-67 expression	
≥15%	28 (84.85%)
<15%	5 (15.15%)
Histological grading	
I	1 (3.03%)
II	18 (54.55%)
III	14 (42.42%)
*De novo* metastasis	
Yes	14 (42.42%)
No	19 (57.58%)
ECOG scores	
1	10 (30.30%)
2	8 (24.24%)
3	15 (45.46%)
Treatment after BMM	
Chemotherapy	18 (54.25%)
Targeted therapy	2 (6.06%)
Endocrine therapy	13 (39.39%)
CDK4/6+AI	9 (27.28%)
AI	4 (12.12%)
Number of lines of therapy received before bone marrow metastases	
0	12 (36.36%)
1	7 (21.22%)
≥2	14 (42.42%)
Combined with bone metastases	
Yes	33 (100%)
No	0 (0%)
Clinical manifestations	
Fatigue	33 (100%)
Fever	25 (75.75%)
Blood routine	
**Pancytopenia**	2 (6.25%)
**Decreased hemoglobin (g/L)**	32 (100%)
100–80	18 (54.55%)
<80	15 (45.45%)
**Thrombocytopenia (10 ^9^/L)**	4 (12.12%)
75–99	3 (9.09%)
<75	1 (3.03%)

### Prognosis

**Figure 2:**
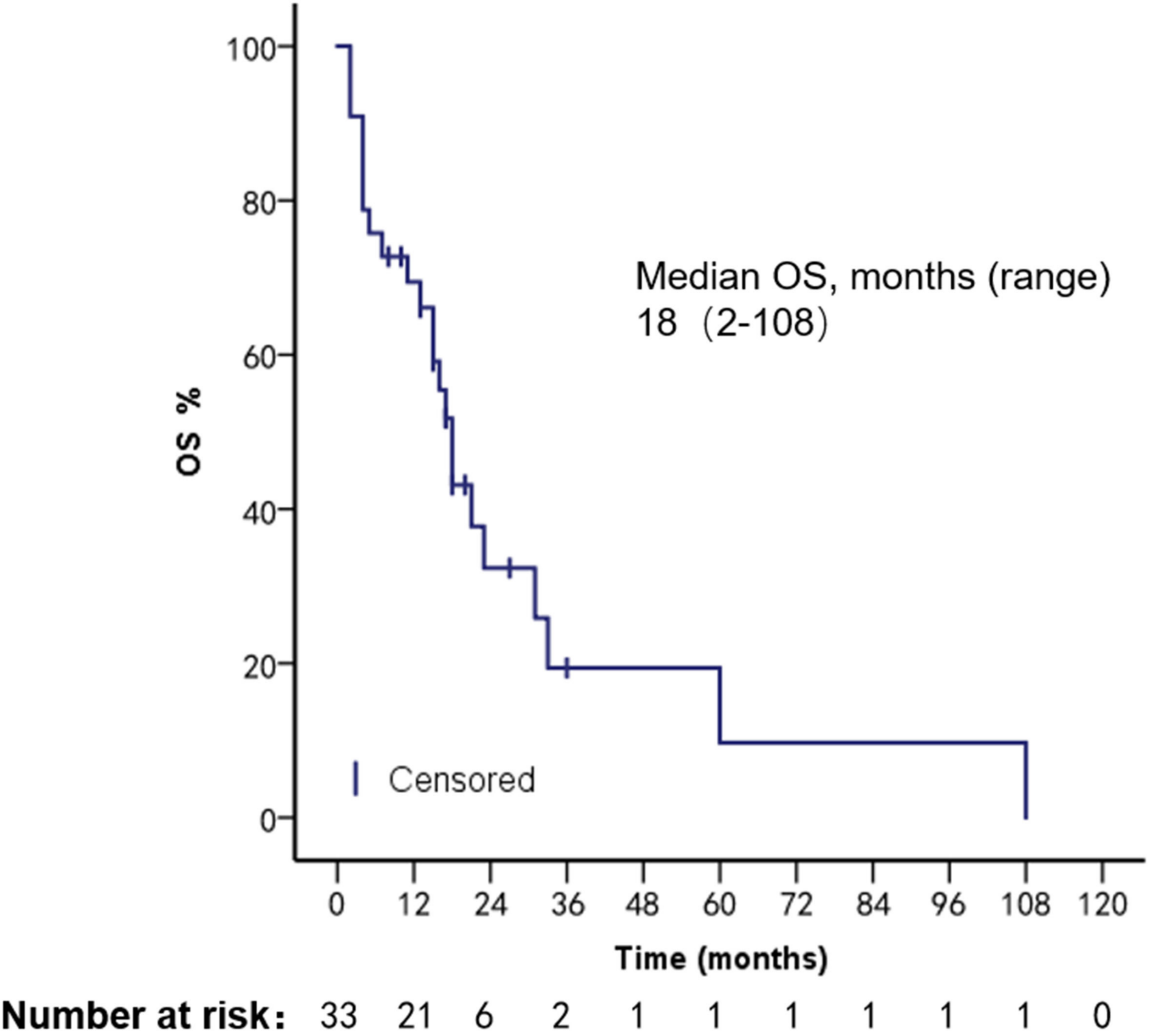
PFS in patients with bone marrow metastases.

**Figure 3:**
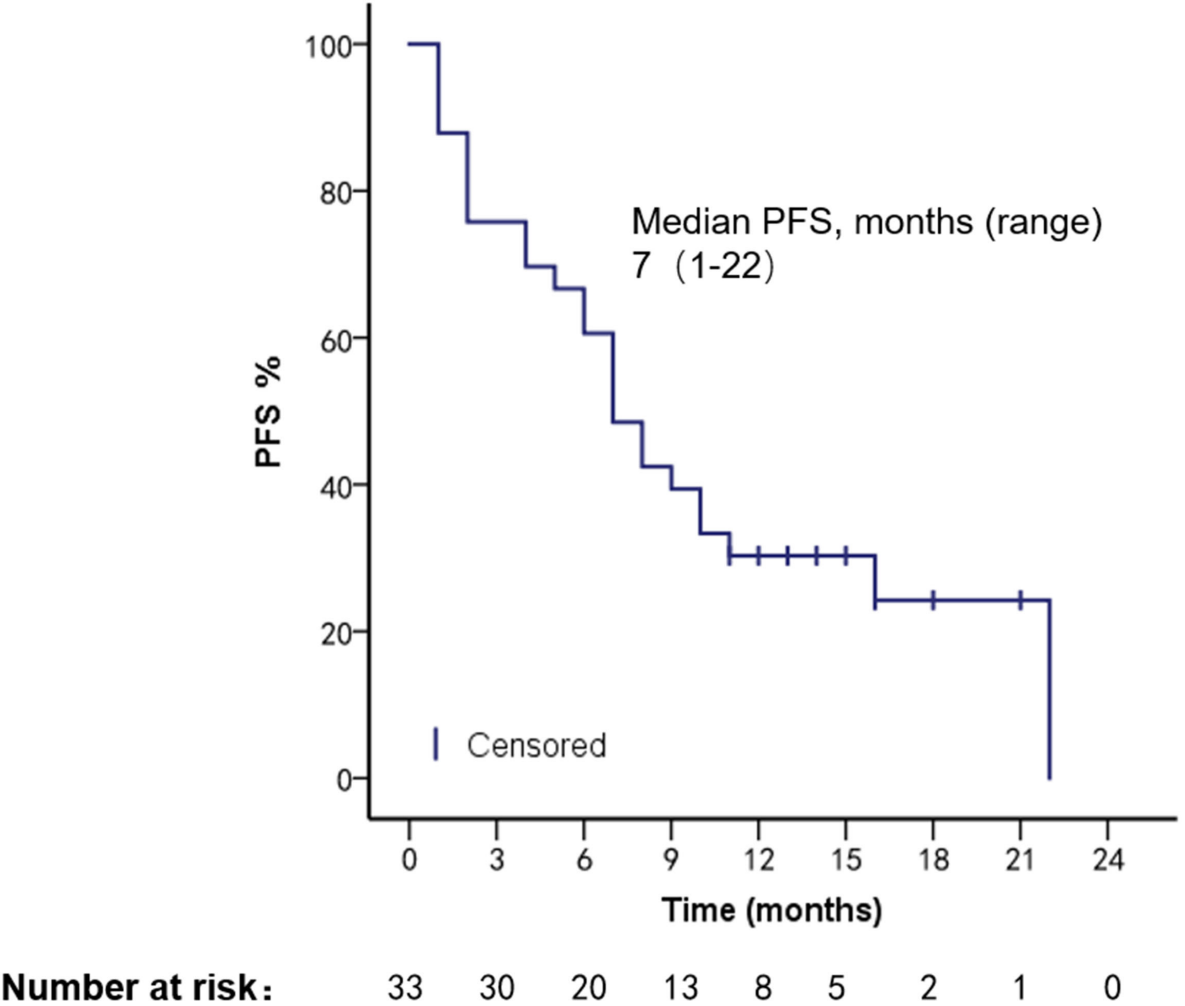
OS in patients with bone marrow metastases.

**Figure 4:**
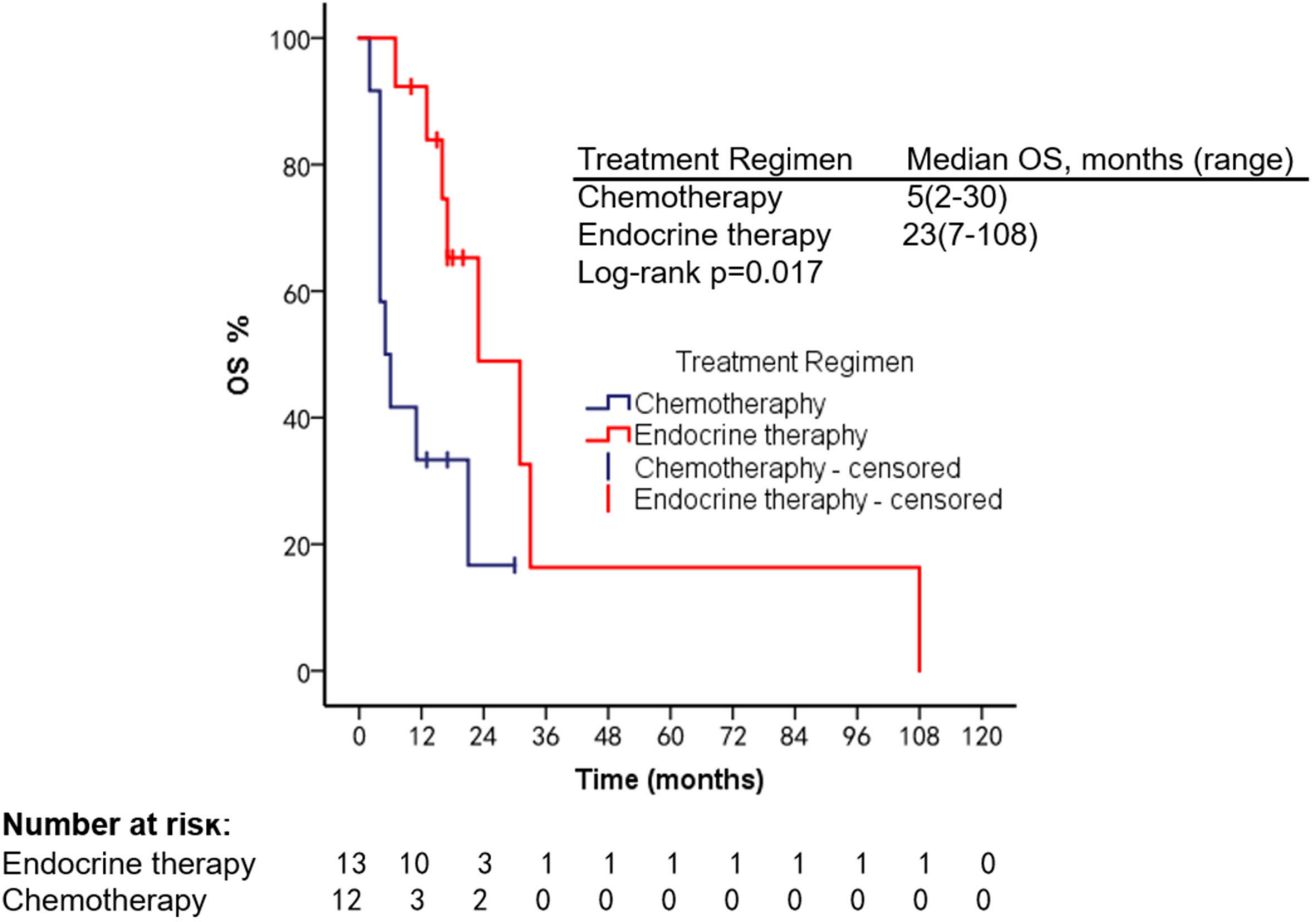
Comparison of PFS in HR^+^/HER2^−^ patients used different treatment regimens.

**Figure 5:**
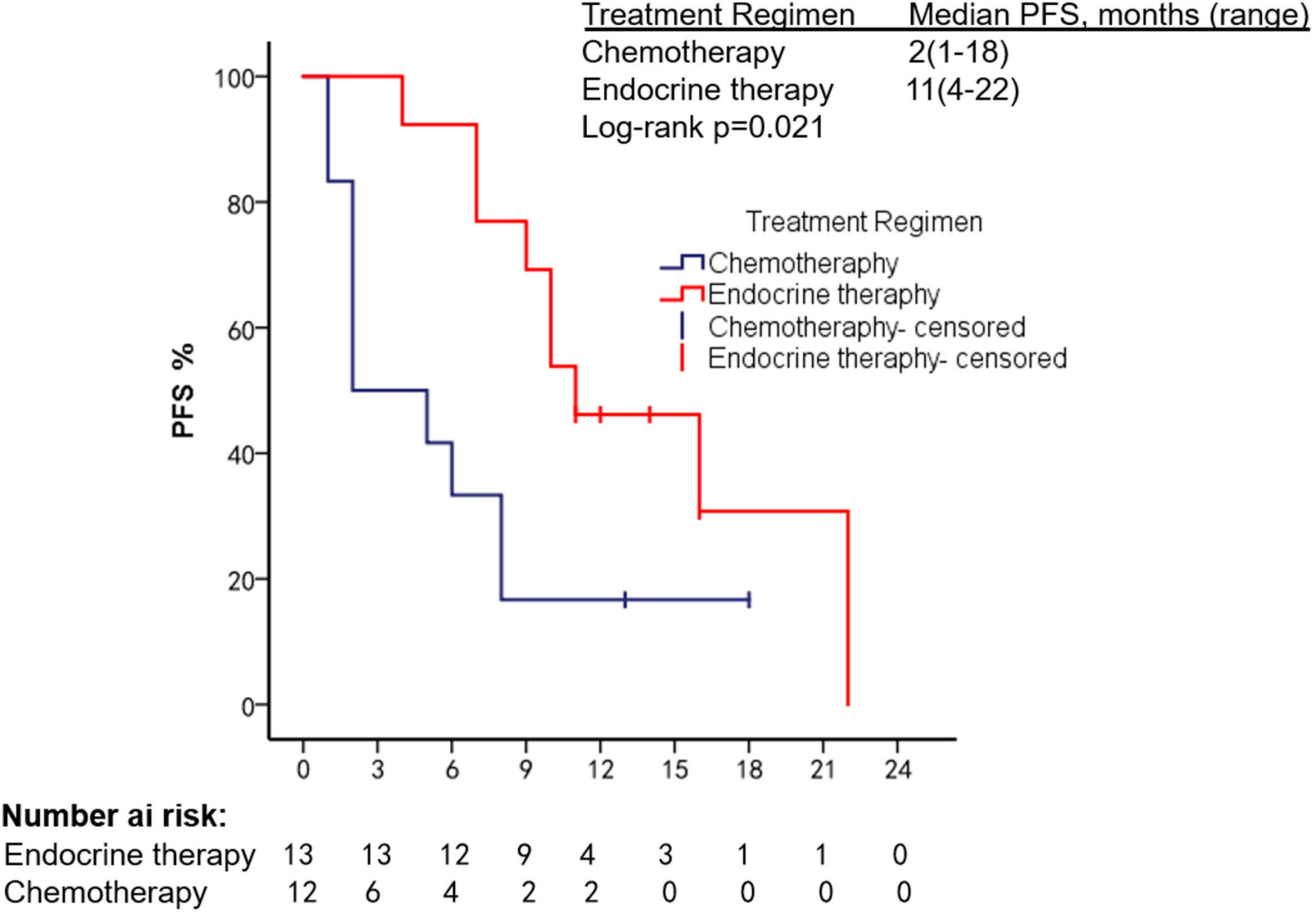
Comparison of OS in HR^+^/HER2^−^ patients using different treatment regimens.

The patients had a median PFS of 7 months (1–22 months) and a median OS of 18 months (2–108 months). We used different treatment regimens to compare the OS and PFS of 25 patients with HR positive/HER2 negative. The median OS for chemotherapy was 5 months (2–30 months), while the median OS for ET was 23 months (7–108 months). The median PFS for chemotherapy was 2 months (1–18 months), and the median PFS for ET was 11 months (4–22 months). Due to the small number of HER2 overexpressing and triple-negative patients, no survival analysis was performed (**Figures 2**–**5**).

In order to investigate the factors affecting the prognosis of patients with BMM, we incorporated elements such as age, menstrual status, initial diagnosis stage, histological grade, ECOG score, and Ki-67 index into the Cox proportional hazards model. We found that a higher ECOG score (95% CI: 2.15–29.28, *p* = 0.002) and older age (95% CI: 2.57–210.84, *p* = 0.005) were associated with shorter survival (**Table 2**).

**Table 2:** Multivariate Cox-regression of overall survival from time of bone marrow metastasis.

Factor	Number (%)	Median OS (months, 95%CI)	Hazard ratio value (95%CI)	*p*-value
Age (years)				
≥60	25, 78.79%	15 (9.0–27.0)	23.27 (2.57–210.84)	**0.005**
<60	7, 21.21%	3 (5.6–21.1)		
Menstrual status				
Postmenopause	11, 33.33%	15 (3.4–37.8)	0.22 (0.047–1.039)	0.056
Premenopausal	22, 66.67%	17 (10.4–22.8)		
Molecular typing				
HR^+^/HER2^−^	25, 75.75%	15.5 (7.9–28.7)	0.979 (0.532–1.802)	0.945
HER2 overexpression	3, 9.09%	15 (−7.2–40.5)		
Triple negative	5, 15.15%	15 (0.68–21.3)		
Ki-67 expression				
≥15%	28, 84.85%	15 (9.0–19.4)	1.721 (0.29–10.09)	0.547
<15%	5, 15.15%	13 (23.0–84.0)		
Histological grading				
1	1, 3.03%	NA	0.398 (0.11–1.44)	0.162
2	18, 54.55%	15 (8.9–17.2)		
3	14, 42.42%	15.5 (4.2–38.7)		
ECOG scores				
1	10, 30.30%	17 (6.7–44.8)	7.940 (2.15–29.28)	**0.002**
2	8, 24.24%	4 (0.6–13.6)		
3	15, 45.46%	4 (5.5–24.7)		
*De novo* metastasis				
Yes	14, 42.42%	15 (5.0–25.3)	1.998 (0.41–9.52)	0.385
No	19, 57.58%	16 (6.9–28.6)		
Pathological type				
Invasive ductal carcinoma	28, 84.85%	15 (8.6–18.4)	1.359 (0.27–6.83)	0.71
Invasive lobular carcinoma	2, 6.06%	NA		
Papillary carcinoma	3, 9.09%	1.5 (12.5–25.5)		
Number of previous treatment lines				
0	12, 36.36%	16 (1.7–38.5)	1.10 (0.47–2.57)	0.82
1	7, 21.22%	15 (4.8–19.1)		
≥2	14, 42.42%	15 (6.3–26.2)		
Hemoglobin level (g/L)				
75–99	18, 54.55%	12 (6.9–21.2)	1.49 (0.38–5.70)	0.56
<75	15, 45.45%	13 (4.8–35.7)		

OS, Overall Survival; CI, Confidence Interval; NA, Not Available.

### Adverse events

Adverse events (AEs) are summarized in **Table 3**. The most common hematologic adverse event in the chemotherapy group was hemoglobin reduction (61.11%), and 15 (83.33%) patients had AEs of grade ≥3. Alopecia was the most common nonhematologic AE (100%) in the chemotherapy group. The common hematologic AEs in the ET group were neutropenia (38.46%) and decrease in hemoglobin (38.46%), and only two (15.38%) patients had grade ≥3 AEs. No patient had a grade 1 hematologic adverse event.

**Table 3:** Statistics of adverse events after treatment (CTCAE 5.0).

	Chemotherapy (*n* = 18)			Endocrine therapy (*n* = 13)		
	Grade 1	Grade 2	Grade ≥3	Grade 1	Grade 2	Grade ≥3
Hematologic AEs						
Neutropenia	0 (0%)	4 (22.22%)	1 (5.56%)	0 (0%)	5 (38.46%)	0 (0%)
Leukopenia	0 (0%)	4 (22.22%)	1 (5.56%)	0 (0%)	0 (0%)	1 (7.69%)
Decreased hemoglobin	0 (0%)	4 (22.22%)	7 (38.89%)	0 (0%)	4 (30.77%)	1 (7.69%)
Thrombocytopenia	0 (0%)	3 (16.67%)	6 (33.33%)	0 (0%)	2 (15.38%)	0 (0%)
Nonhematologic AEs						
Hair loss	8 (44.44%)	10 (55.56%)	0 (0%)	0 (0%)	0 (0%)	0 (0%)
Fatigue	7 (38.89%)	3 (16.66%)	1 (5.55%)	0 (0%)	6 (46.15%)	0 (0%)
Nausea and vomiting	5 (27.78%)	4 (22.22%)	0 (0%)	0 (0%)	0 (0%)	0 (0%)
**AEs result in dose reduction**	6 (33.33%)			2 (15.38%)		

Fatigue was the most common nonhematologic AE (46.15%) in the ET group. Six (33.33%) patients in the chemotherapy group and two (15.38%) in the ET group had dose reductions due to AEs. No patients experienced treatment-related serious adverse events (SAEs). SAEs are life-threatening or fatal events that require hospitalization or prolonged hospitalization, result in permanent or significant disability/loss of function, and congenital anomaly or congenital disability.

AEs are graded according to the Common Terminology Criteria for Adverse Events (CTCAE) Version 5.0.

## Discussion

Clinically relevant bone marrow carcinomatosis (that causes severe cytopenia) is a rare event in patients with breast cancer, with a reported incidence of only 0.17% (2). We retrospectively reviewed 733 patients with advanced breast cancer and found that 33 (4.5%) developed bone marrow metastases, which is higher than previously reported studies and may be related to aggressive bone marrow biopsy and vigilance for patients presenting with anemia and unexplained febricity. Our study found that the median age of patients with bone marrow metastases was 49.5 years old (29–68 years), the pathological type was invasive ductal carcinoma, the molecular type was HR positive/HER2 negative, the Ki-67 index was highly expressed, and histological grades 2–3 were more common, which is similar to the clinical features of breast cancer patients with BMM in a retrospective study by Abdullah Sakin et al. (2, 7). All our patients’ clinical manifestations presented with fatigue, consistent with reported studies (11, 12). There were 25 patients with fever; we performed relevant tests for etiology, and all were negative. Empirical antibiotic therapy did not significantly improve the patient’s symptoms, and immediately we performed a bone marrow aspiration biopsy which revealed BMM. Xiao et al. also reported the phenomenon of unexplained febricity in BMM patients with solid tumors (3). We found that all patients had BMM accompanied by bone metastases. The reported articles did not analyze the specific sites of bone metastases. We found that the weight-bearing bone (spine, 26 cases) was the most common site of metastasis, which may be because the level of CXC3L1/CXC3R1 in the spine bone is higher than that in other bones, which can promote the adhesion and migration of breast cancer cells (13).

We summarized the literature on breast cancer BMM retrieved from PubMed (**Table 4**). The published kinds of literature are mainly case reports. After BMM, chemotherapy is the first choice (11, 14–16). Although some patients’ diseases were controlled, some reports mentioned that chemotherapy-related adverse events led to dose reduction or even discontinuation (11). Turner et al. found that CDK4/6 inhibitor combined with ET can significantly improve the survival of hormone receptor-positive breast cancer patients with visceral metastases (not visceral crisis) (19). Giovanna Garufi et al. reported a patient with hormone receptor-positive breast cancer with BMM who received letrozole in combination with palbociclib and leuprolide and achieved a 26-month sustained complete remission (17). Sakin et al. retrospectively studied 30 patients with breast cancer BMM who had received chemotherapy and had a median OS of only 6 months (7), which is shorter than the median OS of 18 months in our study. Among the 25 patients with HR^+^/HER2^−^ in our research, we were pleasantly surprised to find that the median PFS of 13 patients treated with endocrine therapy was 11 months, which was significantly better than the chemotherapy group of 2 months (Log-rank *p* = 0.021). The maximum PFS was 22 months, and the patient had progressive disease at our follow-up cutoff. The median OS was 23 months longer than the chemotherapy group (5 months). We found that the prognosis of patients in the ET group was significantly improved. The multivariate Cox regression results found that higher ECOG scores and higher age were risk factors affecting the OS of patients, which was consistent with the reported results (2). We counted the AEs after the patients received the two treatment regimens and found that the incidence of AEs of grades≥3 in patients receiving ET was significantly lower than in the chemotherapy group. The proportion of patients with dose reductions due to AEs was also lower in the ET group. Furthermore, no treatment-related SAEs occurred in either group. Indicates that ET may become the most effective and safest treatment option for HR^+^/HER2 patients with BMM, and the sample size should be expanded for further research in the future.

**Table 4:** Summary of clinical characteristics and prognosis of reported breast cancer BMM.

Type of study	Sample	Age/median age	Pathological type	Molecular typing	Histological grading	Treatment	OS	Results
Retrospective study	30	44.5	Invasive ductal carcinoma: 27 Invasive lobular carcinoma: 3	Triple negative: 4 HR positive, HER2 negative: 21 HER2 overexpression: 4	II: 17 III: 13	Chemotherapy	6 months	Chemotherapy significantly prolongs survival in breast cancer patients with bone marrow metastases. Among them, paclitaxel treatment achieved the best survival rate (7).
Retrospective study	22	47	Invasive ductal carcinoma: 14 Invasive lobular carcinoma: 7 Missing: 1	HR positive, HER2 negative: 18 HER2 overexpression: 1 Triple negative: 3	III: 10 II: 8 Missing: 4	Chemotherapy	11 months	Breast cancer patients with bone marrow metastases should receive rescue therapy with a high response rate (2).
Case report	1	62	Invasive ductal carcinoma	HR positive, HER2 negative	III	Chemotherapy	57 months	Aggressive standard-dose chemotherapy may be feasible and beneficial in selected patients with bone marrow cancer-related severe thrombocytopenia without major bleeding events (14).
Pilot study	5	47	–	HR positive, HER2 negative: 3 Missing: 2	–	Palliative hormone therapy combined with low-dose chemotherapy.	12–38 months	Low-dose chemotherapy and oral or intravenous bisphosphonates prolong survival in patients with bone marrow metastases (15).
Case report	1	58	Occult breast cancer	–	–	Symptomatic treatment	–	Bone marrow aspirate has essential implications for diagnosing rare OBC patients (12).
Case report	5	66	Invasive ductal carcinoma: 4 Invasive lobular carcinoma: 1	HR positive, HER2 negative: 5	–	Chemotherapy	19 months	For capecitabine as a treatment option for patients with breast cancer and bone marrow metastases, a study involving many patients is warranted (16).
Case report	1	62	Invasive lobular carcinoma	HR positive, HER2 negative	–	Chemotherapy	44 months	In patients with bone marrow metastases with a good PS score, medical therapy is a consideration (8).
Case report	1	62	Invasive lobular carcinoma	HR positive, HER2 negative	–	Chemotherapy	38 months	Breast cancer metastases to the bone marrow can be life-threatening, and chemotherapy improves survival (11).
Case report	1	41	Invasive ductal carcinoma	HER2 overexpression	–	Trastuzumab	11 months	Trastuzumab may be a beneficial treatment option for patients with HER2-positive bone marrow metastases (10).
Case report	1	46	Invasive lobular carcinoma	HR positive, HER2 negative	–	Palbociclib+letrozole+ovarian suppression	26 months	A combination of endocrine therapy and CDK4/6 inhibitor may have more extended clinical benefits than chemotherapy, and combination therapy of ET and CDK4/6 inhibitor is less toxic and leads to a better quality of life than chemotherapy (17).
Case report	1	58	–	HR positive, HER2 negative	–	Aromatase inhibitor	7 months	After hormonal treatment with an aromatase inhibitor. The patient’s condition improved (18).

To the best of our knowledge, a larger number of patients with symptomatic BMM were included in this study. The clinical characteristics, prognosis, and adverse events were described in detail. We believe that our current study represents the first thorough evaluation of the efficacy and safety of CDK4/6 inhibitor combined with ET applied to patients with symptomatic BMM and provides valuable information for optimizing therapy.

However, there are limitations to this study. Given the limitations inherent to a retrospective, single-center, small sample size study associated with the challenges in identifying patients considered to be in BMM, our results need to be validated in appropriately designed prospective multicenter prognostic studies and clinical trials comparing different treatment modalities for patients with this condition.

## Conclusion

When breast cancer patients present with anemia and fever despite a negative etiological test, BMM should take this into account. For HR^+^/HER2^−^ patients with good physical status and can receive active treatment, CDK4/6 inhibitors combined with endocrine therapy can be used to control disease progression, improve quality of life, and prolong survival.

## Data availability statement

The raw data supporting the conclusions of this article will be made available by the authors, without undue reservation.

## Ethics statement

Ethical review and approval was not required for the study on human participants in accordance with the local legislation and institutional requirements. Written informed consent for participation was not required for this study in accordance with the national legislation and the institutional requirements.

## Author contributions

RY: writing—original draft; review and editing. GL: writing—review and editing. ZL: writing—review and editing. LJ: methodology, funding acquisition, writing—review and editing. JC: conceptualization, methodology, supervision, writing—original draft; review and editing. All authors contributed to the article and approved the submitted version.

## References

[1] Khan S Awan SA Jahangir S Kamran S Ahmad IN. Bone marrow metastasis in clear cell renal cell carcinoma: A case study. Cureus (2019) 11(3):e4181. doi: 10.7759/cureus.4181 31106081 PMC6504026

[2] Kopp HG Krauss K Fehm T Staebler A Zahm J Vogel W. Symptomatic bone marrow involvement in breast cancer–clinical presentation, treatment, and prognosis: A single institution review of 22 cases. Anticancer Res (2011) 31(11):4025–30. 22110237

[3] Xiao L Luxi S Ying T Yizhi L Lingyun W Quan P. Diagnosis of unknown nonhematological tumors by bone marrow biopsy: a retrospective analysis of 10,112 samples. J Cancer Res Clin Oncol (2009) 135(5):687–93. doi: 10.1007/s00432-008-0503-2 PMC12160163 18956213

[4] Luo G He Y Yu X. Bone marrow adipocyte: An intimate partner with tumor cells in bone metastasis. Front Endocrinol (Lausanne) (2018) 9:339. doi: 10.3389/fendo.2018.00339 30013512 PMC6036292

[5] Shin E Koo JS. The role of adipokines and bone marrow adipocytes in breast cancer bone metastasis. Int J Mol Sci (2020) 21(14):4967. doi: 10.3390/ijms21144967 32674405 PMC7404398

[6] Walker ND Patel J Munoz JL Hu M Guiro K Sinha G. The bone marrow niche in support of breast cancer dormancy. Cancer Lett (2016) 380(1):263–71. doi: 10.1016/j.canlet.2015.10.033 26546045

[7] Sakin A Sakalar T Sahin S Yasar N Demir C Geredeli C. Factors affecting survival and treatment efficacy in breast cancer patients with bone marrow metastasis. Breast J (2020) 26(4):815–8. doi: 10.1111/tbj.13647 31562662

[8] Pahouja G Wesolowski R Reinbolt R Tozbikian G Berger M Mangini N. Stabilization of bone marrow infiltration by metastatic breast cancer with continuous doxorubicin. Cancer Treat Commun (2015) 3:28–32. doi: 10.1016/j.ctrc.2014.11.002 25914871 PMC4408922

[9] Cawthorn WP Scheller EL Learman BS Parlee SD Simon BR Mori H. Bone marrow adipose tissue is an endocrine organ that contributes to increased circulating adiponectin during caloric restriction. Cell Metab (2014) 20(2):368–75. doi: 10.1016/j.cmet.2014.06.003 PMC4126847 24998914

[10] Xu L Guo F Song S Zhang G Liu Y Xie X. Trastuzumab monotherapy for bone marrow metastasis of breast cancer: A case report. Oncol Lett (2014) 7(6):1951–3. doi: 10.3892/ol.2014.1999 PMC4049744 24932266

[11] Akagi H Shimada A Chin K Domoto H. Successful stabilization of symptomatic bone marrow metastasis two times in a breast cancer patient. Anticancer Res (2021) 41(6):3139–44. doi: 10.21873/anticanres.15099 34083308

[12] Liu L Zhang J Chen M Ren S Liu H Zhang H. Anemia, and thrombocytopenia as initial symptoms of occult breast cancer with bone marrow metastasis: A case report. Med (Baltimore) (2017) 96(45):e8529. doi: 10.1097/MD.0000000000008529 PMC5690751 29137058

[13] Meng Q Zhou L Liang H Hu A Zhou H Zhou J. Spinespecific downregulation of LAPTM5 expression promotes the progression and spinal metastasis of estrogen receptorpositive breast cancer by activating glutaminedependent mTOR signaling. Int J Oncol (2022) 60(4):47. doi: 10.3892/ijo.2022.5337 35294039 PMC8923652

[14] Bjelic-Radisic V Stöger H Winter R Beham-Schmid C Petru E. Long-term control of bone marrow carcinosis and severe thrombocytopenia with standard-dose chemotherapy in a breast cancer patient: a case report. Anticancer Res (2006) 26(2b):1627–30. 16619583

[15] Freyer G Ligneau B Trillet-Lenoir VV. Palliative hormone therapy, low-dose chemotherapy, and bisphosphonate in breast cancer patients with bone marrow involvement and pancytopenia: Report of a pilot experience. Eur J Internal Med (2000) 11(6):329–33. doi: 10.1016/s0953-6205(00)00121-7 11113657

[16] Ardavanis A Kountourakis P Orphanos G Rigatos G. Low-dose capecitabine in breast cancer patients with symptomatic bone marrow infiltration: a case study. Anticancer Res (2008) 28(1b):539–41. 18383899

[17] Garufi G Carbognin L Orlandi A Palazzo A Tortora G Bria E. The therapeutic challenge of disseminated bone marrow metastasis from HR-positive HER2-negative breast cancer: Case report and review of the literature. Front Oncol (2021) 11:651723. doi: 10.3389/fonc.2021.651723 34692469 PMC8529000

[18] Rahmat C Ikhwan R. Hormonal treatment for symptomatic bone marrow metastasis in breast cancer patients. Maedica (Bucur) (2018) 13(3):238–40. doi: 10.26574/maedica.2018.13.3.238 PMC6290185 30568745

[19] Turner NC Finn RS Martin M Im SA DeMichele A Ettl J. Clinical considerations of the role of palbociclib in the management of advanced breast cancer patients with and without visceral metastases. Ann Oncol (2018) 29(3):669–80. doi: 10.1093/annonc/mdx797 PMC5888946 29342248

